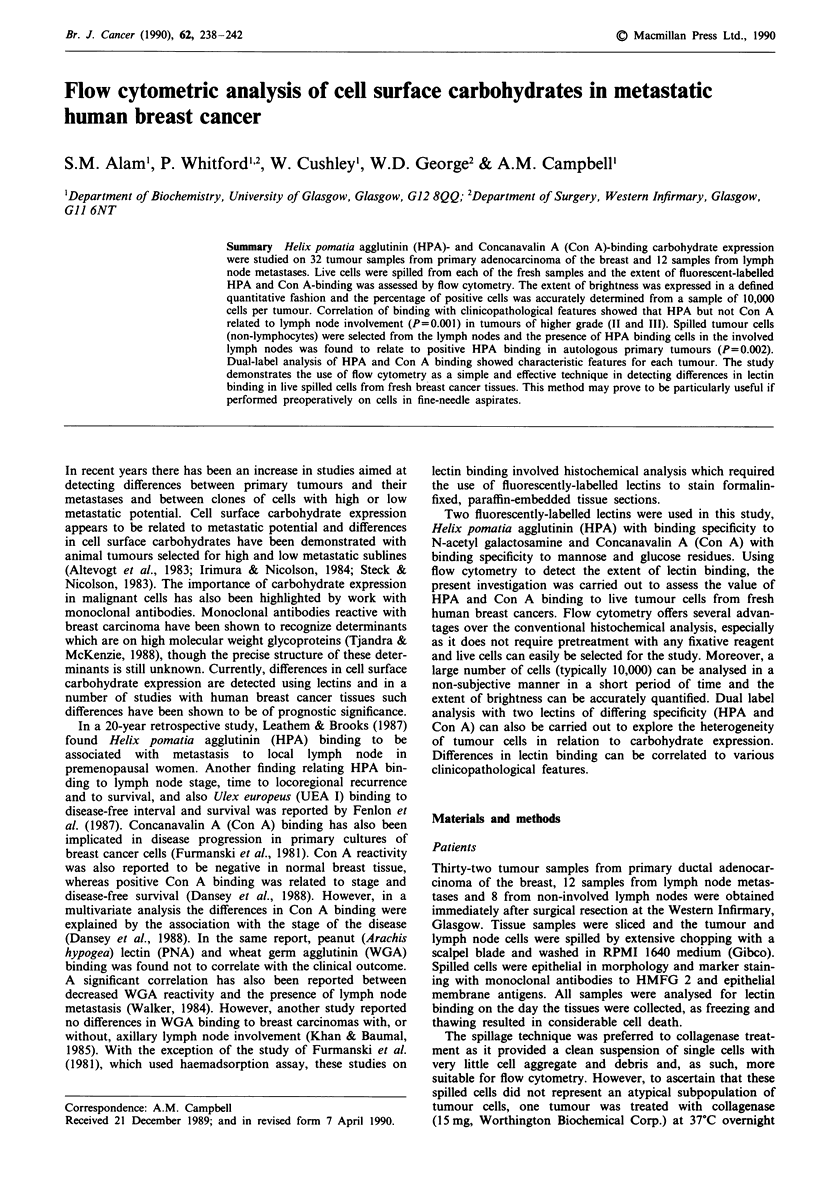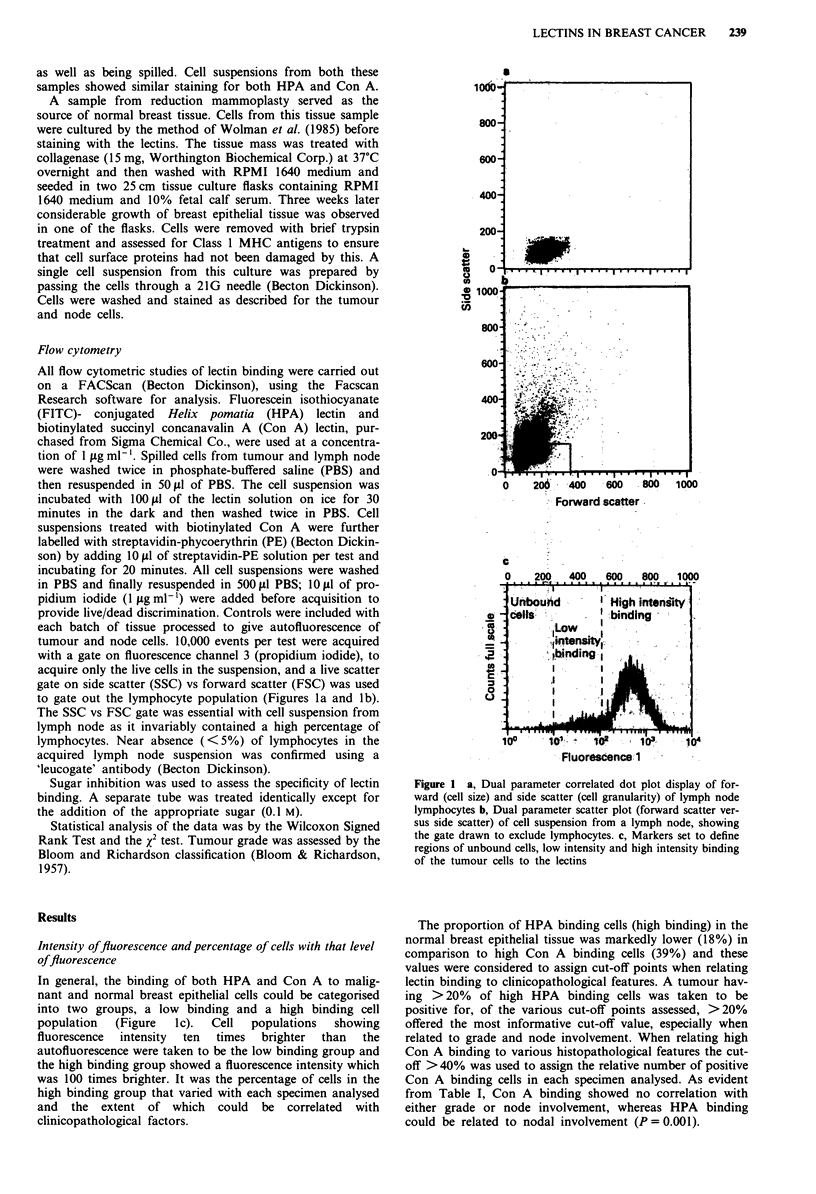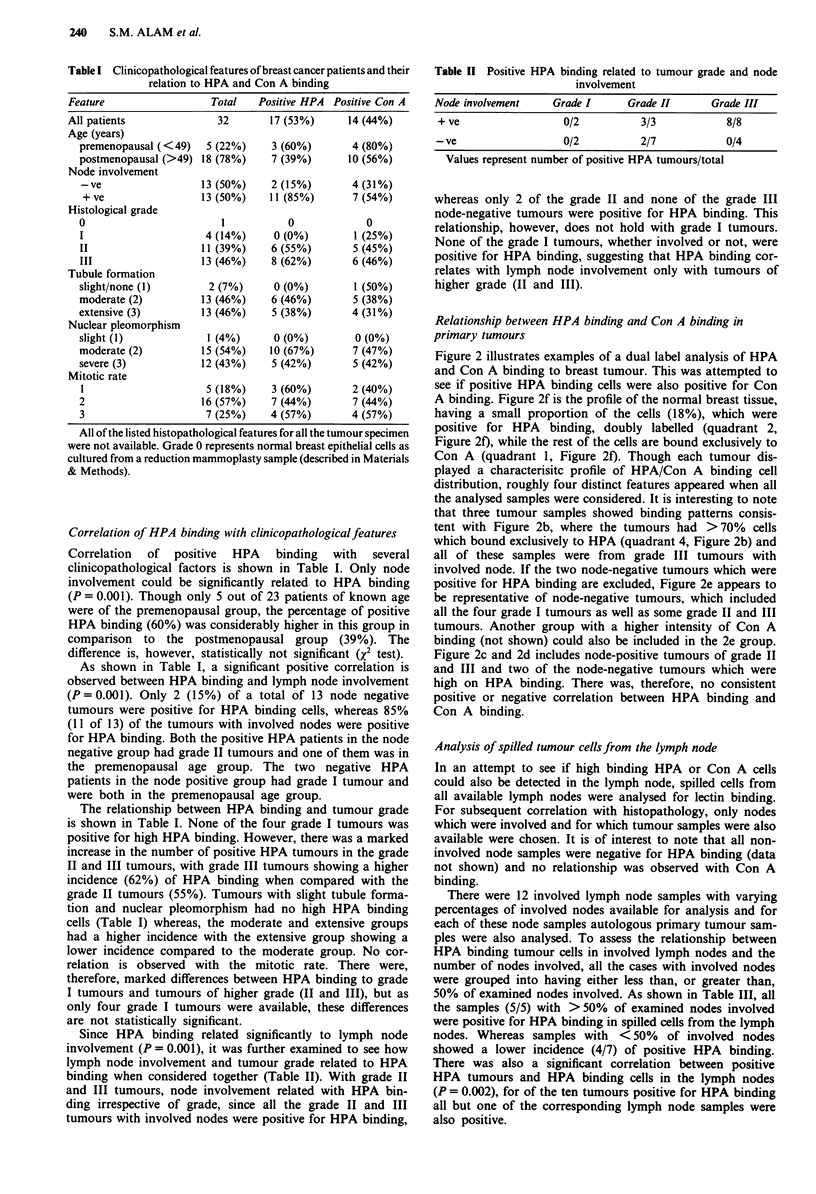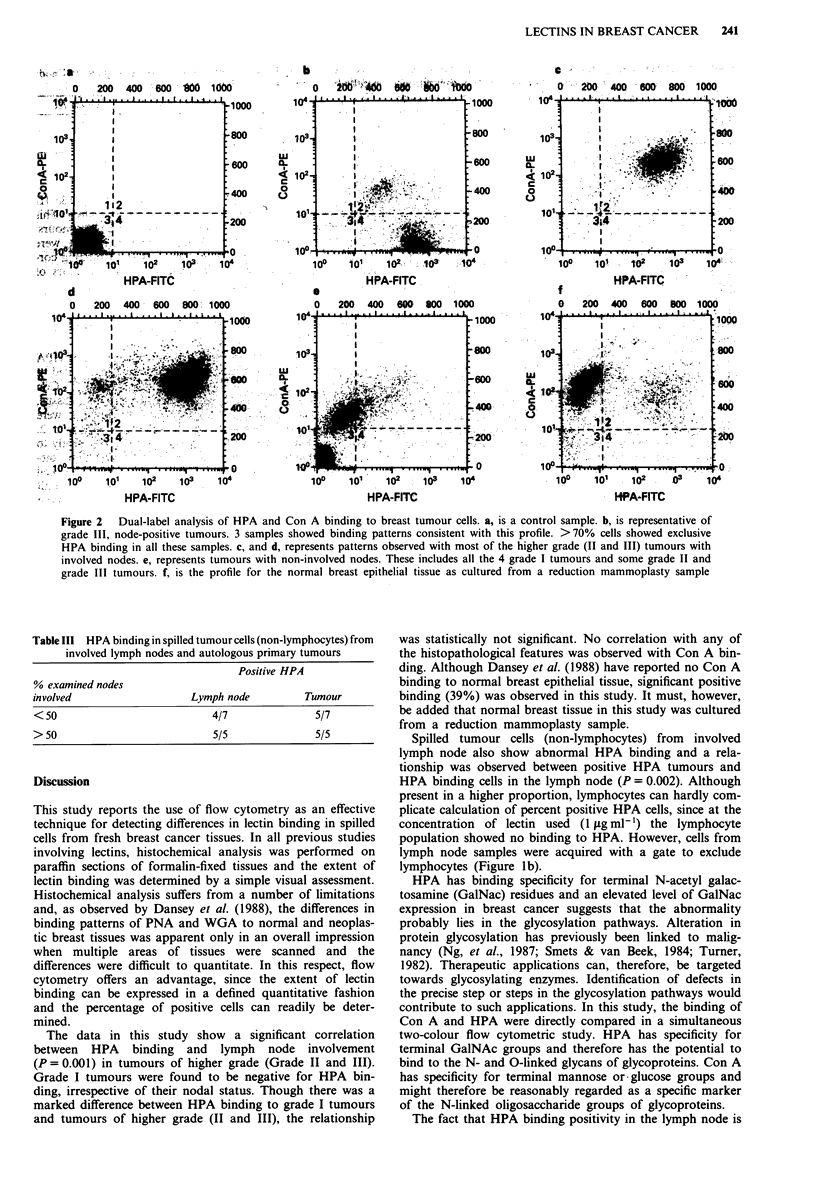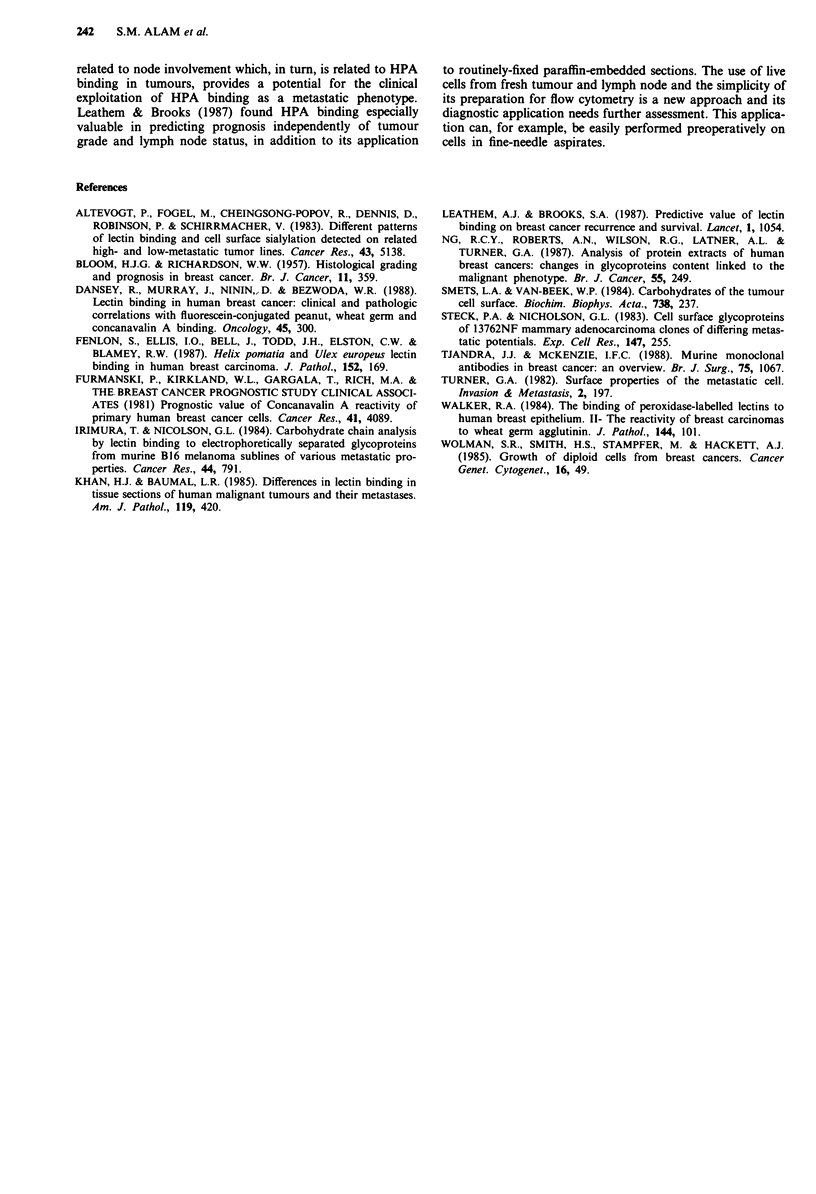# Flow cytometric analysis of cell surface carbohydrates in metastatic human breast cancer.

**DOI:** 10.1038/bjc.1990.267

**Published:** 1990-08

**Authors:** S. M. Alam, P. Whitford, W. Cushley, W. D. George, A. M. Campbell

**Affiliations:** Department of Biochemistry, University of Glasgow.

## Abstract

Helix pomatia agglutinin (HPA)- and Concanavalin A (Con A)-binding carbohydrate expression were studied on 32 tumour samples from primary adenocarcinoma of the breast and 12 samples from lymph node metastases. Live cells were spilled from each of the fresh samples and the extent of fluorescent-labelled HPA and Con A-binding was assessed by flow cytometry. The extent of brightness was expressed in a defined quantitative fashion and the percentage of positive cells was accurately determined from a sample of 10,000 cells per tumour. Correlation of binding with clinicopathological features showed that HPA but not Con A related to lymph node involvement (P = 0.001) in tumours of higher grade (II and III). Spilled tumour cells (non-lymphocytes) were selected from the lymph nodes and the presence of HPA binding cells in the involved lymph nodes was found to relate to positive HPA binding in autologous primary tumours (P = 0.002). Dual-label analysis of HPA and Con A binding showed characteristic features for each tumour. The study demonstrates the use of flow cytometry as a simple and effective technique in detecting differences in lectin binding in live spilled cells from fresh breast cancer tissues. This method may prove to be particularly useful if performed preoperatively on cells in fine-needle aspirates.


Br. J. Cancer (1990), 62, 238–242 © Macmillan Press Ltd., 1990

### Flow cytometric analysis of cell surface carbohydrates in metastatic human breast cancer

S.M. Alam[1], P. Whitford[1,2], W. Cushley[1], W.D. George[2] & A.M. Campbell[1]

[1]*Department of Biochemistry, University of Glasgow, Glasgow, G12 8QQ;* [2]*Department of Surgery, Western Infirmary, Glasgow, G11 6NT*

**Summary** *Helix pomatia* agglutinin (HPA)- and Concanavalin A (Con A)-binding carbohydrate expression were studied on 32 tumour samples from primary adenocarcinoma of the breast and 12 samples from lymph node metastases. Live cells were spilled from each of the fresh samples and the extent of fluorescent-labelled HPA and Con A-binding was assessed by flow cytometry. The extent of brightness was expressed in a defined quantitative fashion and the percentage of positive cells was accurately determined from a sample of 10,000 cells per tumour. Correlation of binding with clinicopathological features showed that HPA but not Con A related to lymph node involvement ($P = 0.001$) in tumours of higher grade (II and III). Spilled tumour cells (non-lymphocytes) were selected from the lymph nodes and the presence of HPA binding cells in the involved lymph nodes was found to relate to positive HPA binding in autologous primary tumours ($P = 0.002$). Dual-label analysis of HPA and Con A binding showed characteristic features for each tumour. The study demonstrates the use of flow cytometry as a simple and effective technique in detecting differences in lectin binding in live spilled cells from fresh breast cancer tissues. This method may prove to be particularly useful if performed preoperatively on cells in fine-needle aspirates.

In recent years there has been an increase in studies aimed at detecting differences between primary tumours and their metastases and between clones of cells with high or low metastatic potential. Cell surface carbohydrate expression appears to be related to metastatic potential and differences in cell surface carbohydrates have been demonstrated with animal tumours selected for high and low metastatic sublines (Altevogt *et al.*, 1983; Irimura & Nicolson, 1984; Steck & Nicolson, 1983). The importance of carbohydrate expression in malignant cells has also been highlighted by work with monoclonal antibodies. Monoclonal antibodies reactive with breast carcinoma have been shown to recognize determinants which are on high molecular weight glycoproteins (Tjandra & McKenzie, 1988), though the precise structure of these determinants is still unknown. Currently, differences in cell surface carbohydrate expression are detected using lectins and in a number of studies with human breast cancer tissues such differences have been shown to be of prognostic significance.

In a 20-year retrospective study, Leathem & Brooks (1987) found *Helix pomatia* agglutinin (HPA) binding to be associated with metastasis to local lymph node in premenopausal women. Another finding relating HPA binding to lymph node stage, time to locoregional recurrence and to survival, and also *Ulex europeus* (UEA I) binding to disease-free interval and survival was reported by Fenlon *et al.* (1987). Concanavalin A (Con A) binding has also been implicated in disease progression in primary cultures of breast cancer cells (Furmanski *et al.*, 1981). Con A reactivity was also reported to be negative in normal breast tissue, whereas positive Con A binding was related to stage and disease-free survival (Dansey *et al.*, 1988). However, in a multivariate analysis the differences in Con A binding were explained by the association with the stage of the disease (Dansey *et al.*, 1988). In the same report, peanut (*Arachis hypogea*) lectin (PNA) and wheat germ agglutinin (WGA) binding was found not to correlate with the clinical outcome. A significant correlation has also been reported between decreased WGA reactivity and the presence of lymph node metastasis (Walker, 1984). However, another study reported no differences in WGA binding to breast carcinomas with, or without, axillary lymph node involvement (Khan & Baumal, 1985). With the exception of the study of Furmanski *et al.* (1981), which used haemadsorption assay, these studies on lectin binding involved histochemical analysis which required the use of fluorescently-labelled lectins to stain formalin-fixed, paraffin-embedded tissue sections.

Two fluorescently-labelled lectins were used in this study, *Helix pomatia* agglutinin (HPA) with binding specificity to N-acetyl galactosamine and Concanavalin A (Con A) with binding specificity to mannose and glucose residues. Using flow cytometry to detect the extent of lectin binding, the present investigation was carried out to assess the value of HPA and Con A binding to live tumour cells from fresh human breast cancers. Flow cytometry offers several advantages over the conventional histochemical analysis, especially as it does not require pretreatment with any fixative reagent and live cells can easily be selected for the study. Moreover, a large number of cells (typically 10,000) can be analysed in a non-subjective manner in a short period of time and the extent of brightness can be accurately quantified. Dual label analysis with two lectins of differing specificity (HPA and Con A) can also be carried out to explore the heterogeneity of tumour cells in relation to carbohydrate expression. Differences in lectin binding can be correlated to various clinicopathological features.

### Materials and methods

### *Patients*

Thirty-two tumour samples from primary ductal adenocarcinoma of the breast, 12 samples from lymph node metastases and 8 from non-involved lymph nodes were obtained immediately after surgical resection at the Western Infirmary, Glasgow. Tissue samples were sliced and the tumour and lymph node cells were spilled by extensive chopping with a scalpel blade and washed in RPMI 1640 medium (Gibco). Spilled cells were epithelial in morphology and marker staining with monoclonal antibodies to HMFG 2 and epithelial membrane antigens. All samples were analysed for lectin binding on the day the tissues were collected, as freezing and thawing resulted in considerable cell death.

The spillage technique was preferred to collagenase treatment as it provided a clean suspension of single cells with very little cell aggregate and debris and, as such, more suitable for flow cytometry. However, to ascertain that these spilled cells did not represent an atypical subpopulation of tumour cells, one tumour was treated with collagenase (15 mg, Worthington Biochemical Corp.) at 37°C overnight

Correspondence: A.M. Campbell
Received 21 December 1989; and in revised form 7 April 1990.

as well as being spilled. Cell suspensions from both these samples showed similar staining for both HPA and Con A.

A sample from reduction mammoplasty served as the source of normal breast tissue. Cells from this tissue sample were cultured by the method of Wolman *et al.* (1985) before staining with the lectins. The tissue mass was treated with collagenase (15 mg, Worthington Biochemical Corp.) at 37°C overnight and then washed with RPMI 1640 medium and seeded in two 25 cm tissue culture flasks containing RPMI 1640 medium and 10% fetal calf serum. Three weeks later considerable growth of breast epithelial tissue was observed in one of the flasks. Cells were removed with brief trypsin treatment and assessed for Class 1 MHC antigens to ensure that cell surface proteins had not been damaged by this. A single cell suspension from this culture was prepared by passing the cells through a 21G needle (Becton Dickinson). Cells were washed and stained as described for the tumour and node cells.

*Flow cytometry*

All flow cytometric studies of lectin binding were carried out on a FACScan (Becton Dickinson), using the Facscan Research software for analysis. Fluorescein isothiocyanate (FITC)- conjugated *Helix pomatia* (HPA) lectin and biotinylated succinyl concanavalin A (Con A) lectin, purchased from Sigma Chemical Co., were used at a concentration of 1 $\mu g\ ml^{-1}$. Spilled cells from tumour and lymph node were washed twice in phosphate-buffered saline (PBS) and then resuspended in 50 $\mu l$ of PBS. The cell suspension was incubated with 100 $\mu l$ of the lectin solution on ice for 30 minutes in the dark and then washed twice in PBS. Cell suspensions treated with biotinylated Con A were further labelled with streptavidin-phycoerythrin (PE) (Becton Dickinson) by adding 10 $\mu l$ of streptavidin-PE solution per test and incubating for 20 minutes. All cell suspensions were washed in PBS and finally resuspended in 500 $\mu l$ PBS; 10 $\mu l$ of propidium iodide (1 $\mu g\ ml^{-1}$) were added before acquisition to provide live/dead discrimination. Controls were included with each batch of tissue processed to give autofluorescence of tumour and node cells. 10,000 events per test were acquired with a gate on fluorescence channel 3 (propidium iodide), to acquire only the live cells in the suspension, and a live scatter gate on side scatter (SSC) vs forward scatter (FSC) was used to gate out the lymphocyte population (Figures 1a and 1b). The SSC vs FSC gate was essential with cell suspension from lymph node as it invariably contained a high percentage of lymphocytes. Near absence (<5%) of lymphocytes in the acquired lymph node suspension was confirmed using a 'leucogate' antibody (Becton Dickinson).

Sugar inhibition was used to assess the specificity of lectin binding. A separate tube was treated identically except for the addition of the appropriate sugar (0.1 M).

Statistical analysis of the data was by the Wilcoxon Signed Rank Test and the $\chi^2$ test. Tumour grade was assessed by the Bloom and Richardson classification (Bloom & Richardson, 1957).

**Figure 1** **a**, Dual parameter correlated dot plot display of forward (cell size) and side scatter (cell granularity) of lymph node lymphocytes **b**, Dual parameter scatter plot (forward scatter versus side scatter) of cell suspension from a lymph node, showing the gate drawn to exclude lymphocytes. **c**, Markers set to define regions of unbound cells, low intensity and high intensity binding of the tumour cells to the lectins

### Results

*Intensity of fluorescence and percentage of cells with that level of fluorescence*

In general, the binding of both HPA and Con A to malignant and normal breast epithelial cells could be categorised into two groups, a low binding and a high binding cell population (Figure 1c). Cell populations showing fluorescence intensity ten times brighter than the autofluorescence were taken to be the low binding group and the high binding group showed a fluorescence intensity which was 100 times brighter. It was the percentage of cells in the high binding group that varied with each specimen analysed and the extent of which could be correlated with clinicopathological factors.

The proportion of HPA binding cells (high binding) in the normal breast epithelial tissue was markedly lower (18%) in comparison to high Con A binding cells (39%) and these values were considered to assign cut-off points when relating lectin binding to clinicopathological features. A tumour having >20% of high HPA binding cells was taken to be positive for, of the various cut-off points assessed, >20% offered the most informative cut-off value, especially when related to grade and node involvement. When relating high Con A binding to various histopathological features the cut-off >40% was used to assign the relative number of positive Con A binding cells in each specimen analysed. As evident from Table I, Con A binding showed no correlation with either grade or node involvement, whereas HPA binding could be related to nodal involvement ($P = 0.001$).

**Table I** Clinicopathological features of breast cancer patients and their relation to HPA and Con A binding

| Feature | Total | Positive HPA | Positive Con A |
|---|---|---|---|
| All patients | 32 | 17 (53%) | 14 (44%) |
| Age (years) | | | |
| premenopausal (<49) | 5 (22%) | 3 (60%) | 4 (80%) |
| postmenopausal (>49) | 18 (78%) | 7 (39%) | 10 (56%) |
| Node involvement | | | |
| – ve | 13 (50%) | 2 (15%) | 4 (31%) |
| + ve | 13 (50%) | 11 (85%) | 7 (54%) |
| Histological grade | | | |
| 0 | 1 | 0 | 0 |
| I | 4 (14%) | 0 (0%) | 1 (25%) |
| II | 11 (39%) | 6 (55%) | 5 (45%) |
| III | 13 (46%) | 8 (62%) | 6 (46%) |
| Tubule formation | | | |
| slight/none (1) | 2 (7%) | 0 (0%) | 1 (50%) |
| moderate (2) | 13 (46%) | 6 (46%) | 5 (38%) |
| extensive (3) | 13 (46%) | 5 (38%) | 4 (31%) |
| Nuclear pleomorphism | | | |
| slight (1) | 1 (4%) | 0 (0%) | 0 (0%) |
| moderate (2) | 15 (54%) | 10 (67%) | 7 (47%) |
| severe (3) | 12 (43%) | 5 (42%) | 5 (42%) |
| Mitotic rate | | | |
| 1 | 5 (18%) | 3 (60%) | 2 (40%) |
| 2 | 16 (57%) | 7 (44%) | 7 (44%) |
| 3 | 7 (25%) | 4 (57%) | 4 (57%) |

All of the listed histopathological features for all the tumour specimen were not available. Grade 0 represents normal breast epithelial cells as cultured from a reduction mammoplasty sample (described in Materials & Methods).

*Correlation of HPA binding with clinicopathological features*

Correlation of positive HPA binding with several clinicopathological factors is shown in Table I. Only node involvement could be significantly related to HPA binding ($P = 0.001$). Though only 5 out of 23 patients of known age were of the premenopausal group, the percentage of positive HPA binding (60%) was considerably higher in this group in comparison to the postmenopausal group (39%). The difference is, however, statistically not significant ($\chi^2$ test).

As shown in Table I, a significant positive correlation is observed between HPA binding and lymph node involvement ($P = 0.001$). Only 2 (15%) of a total of 13 node negative tumours were positive for HPA binding cells, whereas 85% (11 of 13) of the tumours with involved nodes were positive for HPA binding. Both the positive HPA patients in the node negative group had grade II tumours and one of them was in the premenopausal age group. The two negative HPA patients in the node positive group had grade I tumour and were both in the premenopausal age group.

The relationship between HPA binding and tumour grade is shown in Table I. None of the four grade I tumours was positive for high HPA binding. However, there was a marked increase in the number of positive HPA tumours in the grade II and III tumours, with grade III tumours showing a higher incidence (62%) of HPA binding when compared with the grade II tumours (55%). Tumours with slight tubule formation and nuclear pleomorphism had no high HPA binding cells (Table I) whereas, the moderate and extensive groups had a higher incidence with the extensive group showing a lower incidence compared to the moderate group. No correlation is observed with the mitotic rate. There were, therefore, marked differences between HPA binding to grade I tumours and tumours of higher grade (II and III), but as only four grade I tumours were available, these differences are not statistically significant.

Since HPA binding related significantly to lymph node involvement ($P = 0.001$), it was further examined to see how lymph node involvement and tumour grade related to HPA binding when considered together (Table II). With grade II and III tumours, node involvement related with HPA binding irrespective of grade, since all the grade II and III tumours with involved nodes were positive for HPA binding, whereas only 2 of the grade II and none of the grade III node-negative tumours were positive for HPA binding. This relationship, however, does not hold with grade I tumours. None of the grade I tumours, whether involved or not, were positive for HPA binding, suggesting that HPA binding correlates with lymph node involvement only with tumours of higher grade (II and III).

**Table II** Positive HPA binding related to tumour grade and node involvement

| Node involvement | Grade I | Grade II | Grade III |
|---|---|---|---|
| + ve | 0/2 | 3/3 | 8/8 |
| – ve | 0/2 | 2/7 | 0/4 |

Values represent number of positive HPA tumours/total

*Relationship between HPA binding and Con A binding in primary tumours*

Figure 2 illustrates examples of a dual label analysis of HPA and Con A binding to breast tumour. This was attempted to see if positive HPA binding cells were also positive for Con A binding. Figure 2f is the profile of the normal breast tissue, having a small proportion of the cells (18%), which were positive for HPA binding, doubly labelled (quadrant 2, Figure 2f), while the rest of the cells are bound exclusively to Con A (quadrant 1, Figure 2f). Though each tumour displayed a characterisitc profile of HPA/Con A binding cell distribution, roughly four distinct features appeared when all the analysed samples were considered. It is interesting to note that three tumour samples showed binding patterns consistent with Figure 2b, where the tumours had >70% cells which bound exclusively to HPA (quadrant 4, Figure 2b) and all of these samples were from grade III tumours with involved node. If the two node-negative tumours which were positive for HPA binding are excluded, Figure 2e appears to be representative of node-negative tumours, which included all the four grade I tumours as well as some grade II and III tumours. Another group with a higher intensity of Con A binding (not shown) could also be included in the 2e group. Figure 2c and 2d includes node-positive tumours of grade II and III and two of the node-negative tumours which were high on HPA binding. There was, therefore, no consistent positive or negative correlation between HPA binding and Con A binding.

*Analysis of spilled tumour cells from the lymph node*

In an attempt to see if high binding HPA or Con A cells could also be detected in the lymph node, spilled cells from all available lymph nodes were analysed for lectin binding. For subsequent correlation with histopathology, only nodes which were involved and for which tumour samples were also available were chosen. It is of interest to note that all non-involved node samples were negative for HPA binding (data not shown) and no relationship was observed with Con A binding.

There were 12 involved lymph node samples with varying percentages of involved nodes available for analysis and for each of these node samples autologous primary tumour samples were also analysed. To assess the relationship between HPA binding tumour cells in involved lymph nodes and the number of nodes involved, all the cases with involved nodes were grouped into having either less than, or greater than, 50% of examined nodes involved. As shown in Table III, all the samples (5/5) with >50% of examined nodes involved were positive for HPA binding in spilled cells from the lymph nodes. Whereas samples with <50% of involved nodes showed a lower incidence (4/7) of positive HPA binding. There was also a significant correlation between positive HPA tumours and HPA binding cells in the lymph nodes ($P = 0.002$), for of the ten tumours positive for HPA binding all but one of the corresponding lymph node samples were also positive.

**Figure 2** Dual-label analysis of HPA and Con A binding to breast tumour cells. **a**, is a control sample. **b**, is representative of grade III, node-positive tumours. 3 samples showed binding patterns consistent with this profile. >70% cells showed exclusive HPA binding in all these samples. **c**, and **d**, represents patterns observed with most of the higher grade (II and III) tumours with involved nodes. **e**, represents tumours with non-involved nodes. These includes all the 4 grade I tumours and some grade II and grade III tumours. **f**, is the profile for the normal breast epithelial tissue as cultured from a reduction mammoplasty sample

**Table III** HPA binding in spilled tumour cells (non-lymphocytes) from involved lymph nodes and autologous primary tumours

| | *Positive HPA* | |
|---|---|---|
| *% examined nodes involved* | *Lymph node* | *Tumour* |
| <50 | 4/7 | 5/7 |
| >50 | 5/5 | 5/5 |

### Discussion

This study reports the use of flow cytometry as an effective technique for detecting differences in lectin binding in spilled cells from fresh breast cancer tissues. In all previous studies involving lectins, histochemical analysis was performed on paraffin sections of formalin-fixed tissues and the extent of lectin binding was determined by a simple visual assessment. Histochemical analysis suffers from a number of limitations and, as observed by Dansey *et al.* (1988), the differences in binding patterns of PNA and WGA to normal and neoplastic breast tissues was apparent only in an overall impression when multiple areas of tissues were scanned and the differences were difficult to quantitate. In this respect, flow cytometry offers an advantage, since the extent of lectin binding can be expressed in a defined quantitative fashion and the percentage of positive cells can readily be determined.

The data in this study show a significant correlation between HPA binding and lymph node involvement ($P = 0.001$) in tumours of higher grade (Grade II and III). Grade I tumours were found to be negative for HPA binding, irrespective of their nodal status. Though there was a marked difference between HPA binding to grade I tumours and tumours of higher grade (II and III), the relationship was statistically not significant. No correlation with any of the histopathological features was observed with Con A binding. Although Dansey *et al.* (1988) have reported no Con A binding to normal breast epithelial tissue, significant positive binding (39%) was observed in this study. It must, however, be added that normal breast tissue in this study was cultured from a reduction mammoplasty sample.

Spilled tumour cells (non-lymphocytes) from involved lymph node also show abnormal HPA binding and a relationship was observed between positive HPA tumours and HPA binding cells in the lymph node ($P = 0.002$). Although present in a higher proportion, lymphocytes can hardly complicate calculation of percent positive HPA cells, since at the concentration of lectin used ($1\ \mu g\ ml^{-1}$) the lymphocyte population showed no binding to HPA. However, cells from lymph node samples were acquired with a gate to exclude lymphocytes (Figure 1b).

HPA has binding specificity for terminal N-acetyl galactosamine (GalNac) residues and an elevated level of GalNac expression in breast cancer suggests that the abnormality probably lies in the glycosylation pathways. Alteration in protein glycosylation has previously been linked to malignancy (Ng, *et al.*, 1987; Smets & van Beek, 1984; Turner, 1982). Therapeutic applications can, therefore, be targeted towards glycosylating enzymes. Identification of defects in the precise step or steps in the glycosylation pathways would contribute to such applications. In this study, the binding of Con A and HPA were directly compared in a simultaneous two-colour flow cytometric study. HPA has specificity for terminal GalNAc groups and therefore has the potential to bind to the N- and O-linked glycans of glycoproteins. Con A has specificity for terminal mannose or glucose groups and might therefore be reasonably regarded as a specific marker of the N-linked oligosaccharide groups of glycoproteins.

The fact that HPA binding positivity in the lymph node is related to node involvement which, in turn, is related to HPA binding in tumours, provides a potential for the clinical exploitation of HPA binding as a metastatic phenotype. Leathem & Brooks (1987) found HPA binding especially valuable in predicting prognosis independently of tumour grade and lymph node status, in addition to its application to routinely-fixed paraffin-embedded sections. The use of live cells from fresh tumour and lymph node and the simplicity of its preparation for flow cytometry is a new approach and its diagnostic application needs further assessment. This application can, for example, be easily performed preoperatively on cells in fine-needle aspirates.

### References

ALTEVOGT, P., FOGEL, M., CHEINGSONG-POPOV, R., DENNIS, D., ROBINSON, P. & SCHIRRMACHER, V. (1983). Different patterns of lectin binding and cell surface sialylation detected on related high- and low-metastatic tumor lines. *Cancer Res.*, **43**, 5138.

BLOOM, H.J.G. & RICHARDSON, W.W. (1957). Histological grading and prognosis in breast cancer. *Br. J. Cancer*, **11**, 359.

DANSEY, R., MURRAY, J., NININ, D. & BEZWODA, W.R. (1988). Lectin binding in human breast cancer: clinical and pathologic correlations with fluorescein-conjugated peanut, wheat germ and concanavalin A binding. *Oncology*, **45**, 300.

FENLON, S., ELLIS, I.O., BELL, J., TODD, J.H., ELSTON, C.W. & BLAMEY, R.W. (1987). *Helix pomatia* and *Ulex europeus* lectin binding in human breast carcinoma. *J. Pathol.*, **152**, 169.

FURMANSKI, P., KIRKLAND, W.L., GARGALA, T., RICH, M.A. & THE BREAST CANCER PROGNOSTIC STUDY CLINICAL ASSOCIATES (1981) Prognostic value of Concanavalin A reactivity of primary human breast cancer cells. *Cancer Res.*, **41**, 4089.

IRIMURA, T. & NICOLSON, G.L. (1984). Carbohydrate chain analysis by lectin binding to electrophoretically separated glycoproteins from murine B16 melanoma sublines of various metastatic properties. *Cancer Res.*, **44**, 791.

KHAN, H.J. & BAUMAL, L.R. (1985). Differences in lectin binding in tissue sections of human malignant tumours and their metastases. *Am. J. Pathol.*, **119**, 420.

LEATHEM, A.J. & BROOKS, S.A. (1987). Predictive value of lectin binding on breast cancer recurrence and survival. *Lancet*, **1**, 1054.

NG, R.C.Y., ROBERTS, A.N., WILSON, R.G., LATNER, A.L. & TURNER, G.A. (1987). Analysis of protein extracts of human breast cancers: changes in glycoproteins content linked to the malignant phenotype. *Br. J. Cancer*, **55**, 249.

SMETS, L.A. & VAN-BEEK, W.P. (1984). Carbohydrates of the tumour cell surface. *Biochim. Biophys. Acta.*, **738**, 237.

STECK, P.A. & NICHOLSON, G.L. (1983). Cell surface glycoproteins of 13762NF mammary adenocarcinoma clones of differing metastatic potentials. *Exp. Cell Res.*, **147**, 255.

TJANDRA, J.J. & MCKENZIE, I.F.C. (1988). Murine monoclonal antibodies in breast cancer: an overview. *Br. J. Surg.*, **75**, 1067.

TURNER, G.A. (1982). Surface properties of the metastatic cell. *Invasion & Metastasis*, **2**, 197.

WALKER, R.A. (1984). The binding of peroxidase-labelled lectins to human breast epithelium. II- The reactivity of breast carcinomas to wheat germ agglutinin. *J. Pathol.*, **144**, 101.

WOLMAN, S.R., SMITH, H.S., STAMPFER, M. & HACKETT, A.J. (1985). Growth of diploid cells from breast cancers. *Cancer Genet. Cytogenet.*, **16**, 49.